# 4D Multiscale Origami Soft Robots: A Review

**DOI:** 10.3390/polym14194235

**Published:** 2022-10-09

**Authors:** Hyegyo Son, Yunha Park, Youngjin Na, ChangKyu Yoon

**Affiliations:** 1 Department of Mechanical Systems Engineering, Sookmyung Women’s University, Seoul 04310, Korea; 2 Institute of Advanced Materials and Systems, Sookmyung Women’s University, Seoul 04310, Korea

**Keywords:** soft actuators, intelligent systems, self-folding, stimuli-responsive, soft robotics

## Abstract

Time-dependent shape-transferable soft robots are important for various intelligent applications in flexible electronics and bionics. Four-dimensional (4D) shape changes can offer versatile functional advantages during operations to soft robots that respond to external environmental stimuli, including heat, pH, light, electric, or pneumatic triggers. This review investigates the current advances in multiscale soft robots that can display 4D shape transformations. This review first focuses on material selection to demonstrate 4D origami-driven shape transformations. Second, this review investigates versatile fabrication strategies to form the 4D mechanical structures of soft robots. Third, this review surveys the folding, rolling, bending, and wrinkling mechanisms of soft robots during operation. Fourth, this review highlights the diverse applications of 4D origami-driven soft robots in actuators, sensors, and bionics. Finally, perspectives on future directions and challenges in the development of intelligent soft robots in real operational environments are discussed.

## 1. Introduction

Four-dimensional (4D) time-dependent shape transformation is important in soft robotics because it provides effective functional operations (e.g., walking, crawling, gripping, and sensing) with tunable mechanical and chemical properties in the targeted time and place [1,2,3,4,5]. To realize programmable shape changes, origami, a paper-folding art-inspired method associated with self-folding algorithms, has been primarily adapted to transform two-dimensional (2D) planar thin-film templates into three-dimensional (3D) geometries [6,7,8,9,10]. Origami skills have generated extensive interest in art and mathematics communities since the mid 1900’s [8]. Origami paper folding is based on several bending operations performed on a thin sheet without stretching or tearing it to realize complex 2D or 3D geometries [8]. Recently, diverse origami strategies combined with self-folding algorithms have been utilized to transform 2D photo-patterned thin films into 3D structures via folding, bending, wrinkling, or curving without manual aid when exposed to an appropriate input stimulus [11,12,13]. Origami structures can easily achieve complex shape transformations with specific crease patterns in response to external stimuli. In particular, the folding mechanism of origami is used in robotics to realize various structural deformations at length scales, varying from meter to micro/nanometer sizes [14,15,16,17]. In addition, stimuli-responsive polymers, hydrogels, and hybrids have been employed in soft robotics to program shape transformation or self-folding in response to external triggers, such as heat, pH, light, electric, magnetic, and pneumatic signals, as well as in organisms, including bacteria and in DNA [18,19,20,21,22].

From the perspective of the shape transformation of soft robots, after tailoring suitable materials, it is critical to develop manufacturing techniques for fabricating suitable 3D structures. Conventional approaches for creating 3D structures consist of top-down fabrication methods, such as photo- or stereo-lithography, electron beam writing, and replica patterning [7,18,22,23]. For fabricating complex 3D structures, top-down fabrication methods require multi-step integration to construct a broad range of shape-changing soft robots. In addition, direct casting, molding, imprinting, and printing have been widely employed to pattern precise 3D structures [18]. Stimuli-responsive materials combined with 3D or four-dimensional (4D) printing techniques have provided novel and innovative directions towards the development of smart shape-transformable soft robots [24,25,26,27,28].

A broad review of origami-inspired robots was presented for a more comprehensive analysis of the recent advances in robotics [8,9,10,29,30,31,32,33]. This review focuses primarily on the advanced progress in 4D multiscale origami soft robots. The discussion begins with stimuli-responsive materials for designing origami-inspired shape-transformable soft robots. Next, techniques for fabricating stimuli-responsive soft robots are discussed. The third section highlights the recent applications of shape-changing soft robots operated via external triggers. Table 1 categorizes 4D multiscale origami soft robots in terms of parameters including materials, scales, and triggers along with their advantages and disadvantages. In addition, folding mechanisms are discussed using theoretical simulation methodologies. Finally, this article concludes with a summary of the current challenges of 4D shape-transformable soft robots with near-future directions and possible new areas of interest.

## 2. Materials Selection

### 2.1. Hydrogels

**Figure 1:**
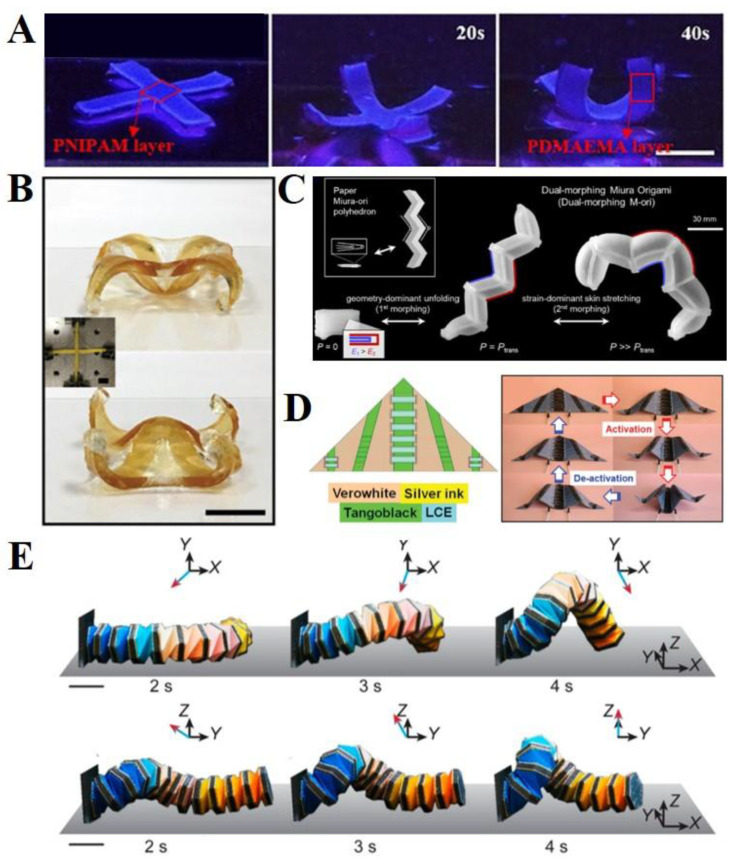
**Soft material-based origami soft robots** (**A**) **Hydrogel:** Dual responsive (thermal and pH variations) hydrogel bilayer. Reproduced with permission [34], copyright 2017, Elsevier B.V. (**B**) **Hydrogel:** Ion-patterned hydrogel origami structure. Reproduced with permission [70], copyright 2018, American chemical Society. (**C**) **Elastomer:** Kinematic composition and dual-morphing behavior of six-module elastomer-based Miura-origami polyhedron. Reproduced with permission [36], AAAS. (**D**) **Liquid crystal:** The activation and deactivation process of the liquid crystal-based airplane. Reproduced with permission [39], copyright 2021, The Royal Society of Chemistry. (**E**) **Paper:** A twisting motion of the paper-based octopus-arm-like robot. Reproduced with permission [49], adapted with permission under the terms of the Creative Commons Attribution Non-Commercial License 4.0, copyright 2021, the authors. Scale bars denote 10 mm (**A**,**B**) and 20 mm (**E**).

Hydrogels have been widely used in the development of time-dependent 4D shape-changing stimuli-responsive soft robots because of their high swelling–deswelling processes in aqueous environments triggered by external cues, such as heat, pH, and light [18,19,20,22,52]. However, because of these local external cues, hydrogels are unsuitable for fabricating large-scale robots. To achieve shape-changing in soft robots, the crosslinking density is mainly considered to affect the physiochemical properties of the lower critical solution temperature (LCST) [53]. Along with the process of swelling (below the LCST)/deswelling (above the LCST), a considerable shape-change of hydrogels has been observed in specified aqueous environments [22,54]. These unique properties of hydrogels can be easily utilized in soft robotics, leading to significant developments in the fields of electronics, sensors, actuators, and optics [5,20,55]. Origami-driven stimuli-responsive hydrogel actuators have been used extensively in soft robotics [13,35,56,57,58,59,60,61]. Among several LCST-based thermally responsive hydrogels, *N*-isopropylacrylamide (NIPAM) has been preferred due to its property of significant swelling/deswelling inside the hydrogel network system, which helps achieve shape-changes in soft robots [11,12,34,62,63,64,65,66]. Cheng et al., presented dual thermal- and pH-responsive origami soft actuators based on a bilayer structure composed of poly(*N*-isopropylacrylamide) (PNIPAM) and poly(2-(dimethylamino) ethyl methacrylate) (PDMAEMA) hydrogels (Figure 1A) [34]. To maximize the actuation response along with the swelling/deswelling process, they tuned the dual stimuli-responsive PNIPAM and PDMAEMA materials to exhibit different physicochemical properties. Upon tuning the unique properties of stimuli-responsive hydrogels, their soft grippers reacted and reconfigured their forms via pH or heat control in DI water and ethanol. Notably, stimuli-responsive hydrogel-based origami robots have been developed for various applications, including biosensors, ionic circuitry, and soft actuators [67,68]. Zhou et al., fabricated complex origami hydrogel actuators using an ionoprinting method [69] to achieve reversible actuation [70]. Specifically, they fabricated origami structures using trivalent ions (Fe^3+^) and tough hydrogels, which consisted of calcium alginate and polyacrylamide (Figure 1B). The stiffness mismatch that exists between the Fe^3+^-embedded and Ca^2+^-embedded pre-stretched hydrogels induces bending actuation at various angles depending on the ratio of Fe^3+^ penetration.

### 2.2. Elastomers

Elastomers are key enablers in the creation of soft robot bodies. Despite their highly nonlinear responses, soft origami robots can actuate to accomplish relatively simple types of motions and tasks that are very difficult to accomplish with hard robots and conventional controllers [47,71,72,73]. In addition, because origami exhibits some plastic deformation, it is difficult to fully recover its basic state. The self-recovery property of elastomers makes them suitable for the deformation of origami robots. [71]. For example, Kim et al., proposed entirely soft dual-morphing architectures that exhibit quasi-sequential behaviors of origami unfolding and skin stretching in response to fluid pressure (Figure 1C) [36]. In particular, they achieved unexplored realms of kinematic features, locomotion, gripping mechanisms, and biomimicry using entirely stretchable elastomers. They suggested a new paradigm for adaptive and extreme shape-morphing systems with high speeds and large magnitudes. Jin et al., presented a vacuum-powered SPA (VSPA) with a Kresling origami block, a type of volumetric origami [71]. The actuator is made of a flexible thermoplastic elastomer (TPE) and can withstand larger payloads, whereas the deformation can be ignored in the unactuated state. Furthermore, the VSPA can achieve twist and contraction motions. In particular, pure contraction can be realized in VSPA through bistability. 

To achieve precise shape change, locomotion, manipulation, function of soft robots, and silicone-based materials have been combined with other functional materials to enhance their multi-responsive and highly sensitive properties [74,75,76]. For example, Martinez et al., used a silicone elastomer and paper to increase the range of motion (e.g., extension, contraction, bending, extension, and torsion) available for soft robots while maintaining a high performance-to-cost ratio. Elastomers combined with paper are lightweight and easy to fabricate. Additionally, paper can be transformed into a range of complex 3D structures [47]. As a replacement for parts made of silicone rubbers, Lin et al., presented origami “skeletons” using four stacks of 12-layer sandpaper with origami creases in the middle of the four folding faces [73]. The flexible sandpaper becomes strongly coupled through friction between the layers of sandpaper when external pressure is applied, and the stiffness can be controlled by adjusting the vacuum pressure of the folding faces. Thus, the “skeletons” selectively possess the flexibility and adaptability of silicon rubbers or high force and the shape-locking ability of sandpaper. 

### 2.3. Liquid Crystal

Liquid crystal polymer networks (LCNs) and liquid crystal elastomers (LCEs) have also been utilized in stimuli-responsive actuators based on origami design [39,75,76,77,78,79,80,81]. In short, liquid crystals are materials with state properties between those of liquids and solid crystals [38]. Specifically, the shape change of LCEs can be pre-programmed by arranging the liquid crystal phase before crosslinking. Liquid crystals can demonstrate shape-memory behavior after their molecular alignments are tuned (e.g., twisted nematic and splay configurations), which are regular or collapsed according to thermal changes [81]. Therefore, LCNs and LCEs are suitable materials for actuators with direct control and deformation by photo-mechanisms, and they can also be a limitation regarding scale-up like hydrogels. Yuan et al., suggested a soft airplane actuator using LCEs and slightly crosslinked liquid crystalline polymer networks, which are typically used for light-responsive actuators (Figure 1D) [39]. When a voltage was applied, the LCEs on the electrode changed their orientation from nematic to isotropic, resulting in the largest shape deformation at a bending angle of 140°. Jiang et al., proposed LCN-based actuators with various 3D shape-transformable structures (e.g., airplanes, bulls, and frogs) [82]. These folded structures were performed using a 2D planar film through a photo reconfiguration process. In addition, Yang et al., utilized various multi-temperature-driven actuation systems composed of two thermally responsive LCNs [75]. They fabricated two thermos-sensitive flower-shaped actuators that changed their shapes sequentially under a heat process from 40 °C to 60 °C.

### 2.4. Shape-Memory Materials

Shape-memory materials are smart materials that can return to their original shape when triggered by an external stimulus, such as a temperature change. Shape-memory polymers (SMPs) [41,42,43,44] and shape-memory alloys (SMAs) [42,43,44,45,46] have been widely used in robotics. The shape-memory property of these materials makes them applicable in numerous fields including biomedical devices, aerospace structures, and morphing structures. In particular, SMAs have been used in lightweight solid-state actuators. However, they are not suitable for applications in medical-implantable devices because most SMAs are permanent and do not degrade in the body. Several types of triggers including (direct) heat, light, and electricity can be utilized to induce this smart advantage. For example, Xing et al., proposed a shape-memory soft actuator with electric/moisture actuation [44]. In particular, a flexible shape-memory polymer (FSMP) was used in combination with the AgNP layer and PVA–AgNP composite for the electrothermal response. Moreover, the application of ultralow voltage (2V) resulted in a fast response (within 10 s) and high stability (1000 folding cycles). In their study, poly (ε-capro-lactone) (PCL) was used as the shape-memory material and was fabricated in film form. In addition, electrical and moisture sources were utilized as triggers. More methods have been developed for the fabrication of shape-memory materials; these include 3D/4D printing and actuation, such as heat and light. These methods will be discussed further in the following sections. 

### 2.5. Hard Sheets

A single square hard sheet can be folded into numerous 3D shapes depending on its folding. An origami robot with hard sheets, such as paper and plastic sheets, has a highly versatile shape-changing capability that does not require a complex assembly process. Creating a folding pattern is a challenge in designing a specific 3D shape, such as multimodal deformations [49,50], and creating flexible origami robots with rigid paper [83,84,85,86]. Wu et al., developed a stretchable origami robotic arm with multimodal deformations (Figure 1E) [49]. This robotic arm can deform by continuous stretching and contraction using the Kresling pattern and can achieve reconfigurable omnidirectional bending and multi-axis twisting. 

Another innovative strategy for fabricating flexible origami robots with hard sheets has also been reported. For example, Chen et al., presented origami energy-storing springs and soft robotic mechanisms for performing controlled, dynamically actuated tasks [83]. The authors leveraged a reconfigurable expanding bistable origami pattern and found that small changes in the cone angle of the fold pattern altered the geometry structure and its rigidity. They used 8 mm thick polyester-coated paper with adhesive transfer tape for gluing. In particular, distortion of the paper and deformation of the crease regions according to the number of folds were identified. As the cyclic deformation proceeded, the paper surpassed the elastic deformation limit, and the crease regions underwent permanent plastic deformation. Therefore, mechanical offset strain remained in the structure. They confirmed that no physical damage was observed after approximately 2000 cycles, and no failure was observed on the force–displacement graph.

## 3. Fabrication

After tailoring suitable materials, the manufacturing methods must be organized to create 4D multiscale origami soft robots. To fabricate soft robots, diverse methodologies have been proposed. These approaches range from macroscale (e.g., manual assembly, molding, printing, and deploying–stacking) to nanoscale (e.g., photolithography, laser writing, nanoimprinting, and bottom-up synthesis). In this section, we briefly focus on recent progress in fabrication strategies in terms of molding, printing, and deploying–stacking to create 4D multiscale origami soft robots.

### 3.1. Molding

**Figure 2:**
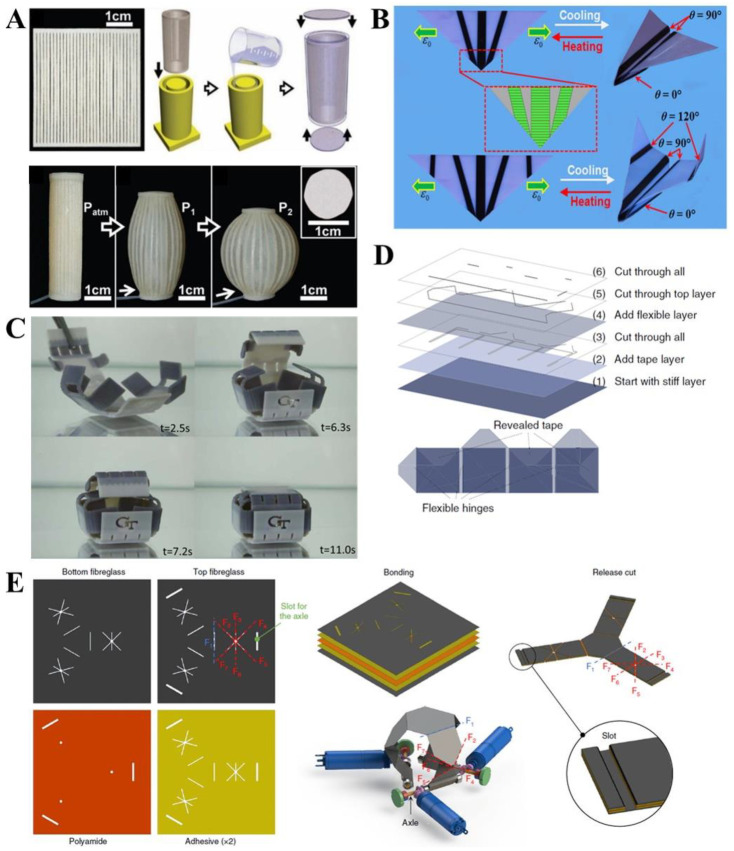
**Diverse fabrication methods of origami soft robots.** (**A**) **Molding:** The 2D pattern of the paper and schematic representation of the molding process. Reproduced with permission [47], copyright 2012, WILEY-VCH. (**B**) **3D/4D printing:** Active origami airplanes. Reproduced with permission [101], copyright 2014, Publishing Ltd. (**C**) **3D/4D printing:** Folding box with self-locking mechanism under heating. Reproduced with permission [102]. Adapted with permission under the terms of the Creative Commons Attribution 4.0 International License, copyright 2015, the authors. (**D**) **Deploying and staking:** Diagram of the three steps to form flat building blocks with both flexible and rigid regions. Reproduced with permission [106]. Adapted with permission under the terms of the Creative Commons Attribution 4.0 International License, copyright 2016, the authors. (**E**) **Deploying and staking:** Foldaway-pushbutton origami robots. Reproduced with permission [108], copyright 2019, Springer Nature.

Direct casting or molding is a widely used method for constructing shape-changing soft robots because of the rapid, inexpensive, and easily accessible processes involved therein [87]. In particular, molding fabrication methods have been extensively employed when using silicone-based soft materials, such as PDMS, Ecoflex, and Dragonskin, to create smart origami structures [88,89]. In addition, silicone-based soft material hybrids with rigid paper [47,90,91,92,93] or different elastomers [36] have displayed another advanced functionality of origami robots using several molding processes. Martinez et al., fabricated pneumatic networks (PneuNets) using an advanced molding fabrication method for composite structures comprising elastomers and flexible sheets (Figure 2A). [47]. To design the PneuNets, a cylindrical mold was designed using 3D printing with ABS plastic. Flexible paper with a crease pattern was rolled into the mold, and the elastomer was poured into the mold following the patterned paper. Kim et al., developed pelican-eel-inspired dual-morphing architectures via repetitive molding, pouring, and curing [36]. They used several elastomers with different stiffnesses, such as Dragonskin 30 and Dragonskin 10, to achieve the required quasi-sequential behavior. The structures were fabricated by sequentially stacking molds according to the desired structures. This fabrication method is suitable for designing elastomer-based structures. However, it is difficult to design complex structures that exhibit multifunctionality.

### 3.2. 3D/4D Printing

This review presents a broad discussion of 3D printing technologies for patterning 3D structures using diverse materials, such as plastics, hydrogels, and even cell-cultured soft materials [94,95]. The 3D printing technology can realize precise spatial placements of many materials in targeted domains or paths with high resolution to automatically create complex 3D structures [95,96,97]. Recently, a variety of time-dependent shape-deformable origami actuators have been proposed using a 4D printing strategy [27,28,98,99,100]. For instance, Ge et al., employed 4D printing technology to fabricate shape-reconfigurable origami airplanes (Figure 2B) [101]. They fabricated diverse shape-transformable 3D structures, from simple (e.g., box and pyramid) shapes to complex (e.g., airplane) shapes. The proposed an origami-driven airplane-shaped actuator comprised of passive panels and active hinges. This origami airplane displayed a wide range of wing movements from 0° to 120°, according to the heating and cooling cycles [101]. Mao et al., proposed active origami structures using 4D printing technologies (Figure 2C) [102]. They fabricated an origami box robot composed of SMPs that displayed sequential folding in a programmable manner. The box robot displayed a locking system realized by placing holes in the hinges and hanging other panels as rings. The thermally responsive origami box robot required 11 s to reversibly display the lock (heating)-and-open (cooling) processes. This method is suitable for fabricating structures with complex designs and compositions; however, there are some limitations regarding the materials utilizable for ink printing. 

### 3.3. Deploying and Stacking

Deployment and stacking strategies have been extensively used to fabricate complex origami structures composed of several materials with diverse crease patterns displayed [103,104]. In short, the deployment and stacking method is primarily utilized to create crease patterns and integrate various material-based patterns [50,105,106,107] or multi-step patterning [108] into a single mechanical system. Using this multi-selective fabrication method, Overvelde et al., fabricated a single extruded cube unit cell (Figure 2D) [106]. Thin-walled unit cells were designed using an efficient staking and deployment method consisting of three layers: two outer layers of polyethylene terephthalate with thicknesses of t = 0.25 mm and t = 0.05 mm and double-sided tape with a thickness of t = 0.05 mm [106]. In particular, because the structural deformation of the unit cell occurs at the hinges, it is fabricated by considering the flexibility of the hinge and rigidity of the faces. Based on the thickness ratio of the face and hinge, the thickness for the appropriate deformation was selected, which was reflected through the stacking method. Additionally, Mintchev et al., developed a foldaway-pushbutton origami mechanism by stacking multiple patterned layers (Figure 2E) [108]. Their origami robot was composed of rigid fiberglass sheets with flexible polyimide hinges. In particular, to realize smart shape-reconfigurable 3D structures from 2D planar sheets, three layers were independently laser-micromachined with deployment and were then stacked sequentially. These origami robots were used as foldaway interfaces for a haptic exploration of the anatomy atlas, handheld joystick, and bimanual controller for drones. The deployment and stacking method is appropriate for designing the multifunctionality of origami robots with rigid materials, such as paper and film. In addition, it is easy to fabricate a modular robot with a hard sheet; therefore, it is possible to combine and expand modules. However, this method is unsuitable for use with fluid actuators such as pneumatic actuators.

## 4. Actuation Triggers

As discussed in the previous sections, there has been enormous interest in origami soft robots that undergo reconfigurable shape changes in response to external stimuli, such as light [59,109,110,111], heat [12,57,67,112,113,114], and pH variation [34,115,116,117,118].

### 4.1. Light

**Figure 3:**
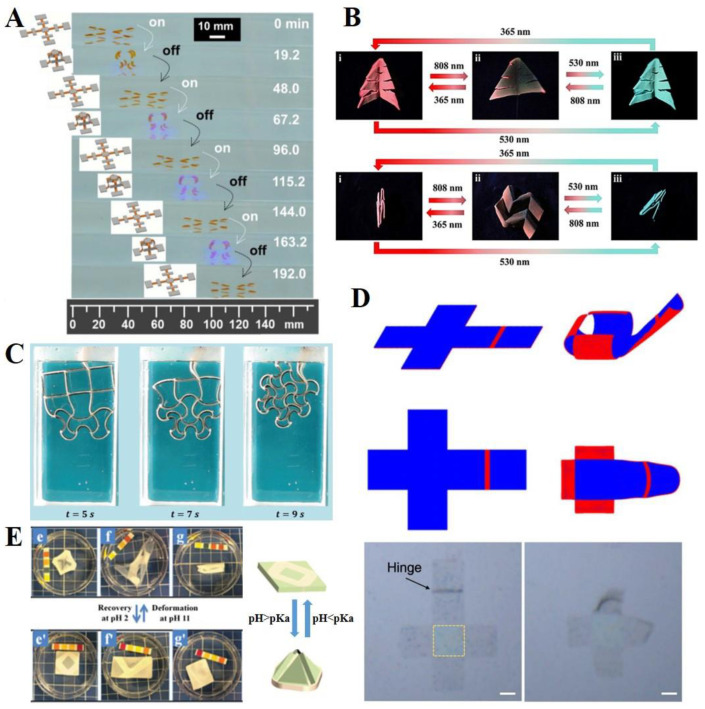
**Diverse external triggers for origami soft robots** (**A**) **Light:** Four walking cycles of origami robots by controlling the light irradiation on-and-off process. Reproduced with permission [35], copyright 2021, Elsevier Inc. (**B**) **Light:** Simultaneous multicolor fluorescence change and complex shape-morphing of a 3D liquid crystal-based soft actuator. Reproduced with permission [79], copyright 2021, Wiley-VCH GmbH. (**C**) **Heat:** The time sequence of the shape transformations in thermally responsive 2D cellular solids. Reproduced with permission [41], adapted with permission under the terms of the Creative Commons Attribution Non-Commercial License 3.0, copyright 2016, the authors. (**D**) **Heat:** Self-folding of thermally responsive ultrathin graphene microstructures. Scale bar means 0.1 mm. Reproduced with permission [114], adapted with permission under the terms of the Creative Commons Attribution Non-Commercial License 4.0, copyright 2017, the authors. (**E**) **pH:** Shape deformation of the hydrogel-based origami robot at pH 11 and pH 2 buffer solutions. Reproduced with permission [118], copyright 2018, The Royal Society of Chemistry.

Light-sensitive materials undergo reversible photomechanical property changes that trigger shape transformation in soft origami robots. There are distinct requirements in terms of the fact that light-responsive materials enable actuators to have relatively instantaneous responses, and they do not require physical contact with the stimulus; however, there are also limitations regarding the response environment, such as transparent space and unblocked area. Therefore, development is necessary if a light-responsive actuator is to be used in vivo. Recently, light-responsive self-folding actuators have been investigated. For example, Li et al., reported a molecular design of photoactive bilayer actuators inspired by an octopus (Figure 3A) [35]. The octopus-shaped actuator was fabricated as a bilayer structure composed of photo-expanding and photo-contracting layers that could maximize bending performance. Layered spiropyran (SP)-based materials have been used in photo-contracting and photo-expanding structures, and they respond simultaneously to a single light stimulus. The octopus-like actuator displayed a smart crawling of approximately 120 mm in the presence (~20 min) and absence (~30 min) of blue light (450 nm). More recently, Aggarwal et al., suggested different states of bending directions to exhibit a more complex actuation of an octopus-shaped actuator [60]. They designed three states of two curved states (+1, −1) and a flat state (0), displaying more than five complicated shape changes from the primary 2D shape. Martella et al., developed a light-responsive liquid crystal-based origami gripper [111]. They used two differently aligned states of liquid crystalline networks (LCNs). Based on the process during the light on/off state, the orientation of the uniformly aligned LCNs changed to irregular states, resulting in shape changes, such as bending, twisting, or folding [81,119]. In addition, light-responsive LCN-based actuators can be operated in diverse environments, such as air or aqueous states [78,120]. Recently, Huang et al., proposed origami-based LCN-tetraphenylethene (TPE)-SP hybrid actuators to achieve complex shape transformations and camouflage (fluorescence change) under three different lights: ultraviolet, visible light, and near infrared (Figure 3B) [79]. The origami actuator showed an unfolding transformation under 808 nm light and a folding transformation under 365 nm light. In addition, it could show both color-changing and shape-morphing behaviors when light of wavelength nm was applied because of the synergic control in the LCE films.

### 4.2. Heat

Thermal triggers have been commonly utilized to actuate origami soft robots, owing to their easy accessibility and versatility. For example, Janbaz et al., adopted thermally responsive SMPs, polyolefins, and hyperplastic bilayer composites to induce complex origami folding and wrinkling [41]. They demonstrated thermally responsive diverse origami soft robots, such as self-twisting DNA-inspired structures, two-dimensional cellular solids (Figure 3C), and self-folding cubes. These origami soft robots can change their shapes within 1 min in the temperature range of 50–80 °C [41]. As mentioned in Section 2, thermally responsive hydrogels are widely used in soft origami actuators. Xu et al., fabricated thermoresponsive self-folding 3D graphene structures combined with PNIPAM hydrogels to develop smart graphene-based origami field-effect transistors with three different origami forms: flowers, dumbbells, and boxes (Figure 3D) [114]. They displayed two folding and unfolding states during heating (45 °C) and cooling (25 °C) cycles, respectively. Generally, direct heating processes are performed in particular environments, such as in aqueous systems. However, secondary sources for conversion into heat may also be required, for example, in the case of in vivo system applications.

### 4.3. pH

The pH-responsive properties have also been widely considered as external stimuli to trigger hydrogel-based soft robots for origami-shape transformations. Hydrogel-based soft robots undergo osmotic pressure-based volume changes from collapsed to expanded, owing to the pH-driven neutral-to-negative charges inside the hydrogel networks [53]. Acrylic acid, methacrylic acid, ethylacrylic acid, and butylacrylic acid are the primary materials used in pH-responsive origami soft robots [121]. Therefore, some limitations can be expected for actuators because they must be used only in pH solutions. Shang et al., utilized these properties and two types of hydrogel to fabricate self-folding quadrangular pyramid structures in response to pH changes (Figure 3E) [118]. They used polyacrylic acid and PNIPAM hydrogels as dual-responsive actuators (thermal and pH responsive). Therefore, they can undergo transformations under temperature changes near the LCST. In addition, this pH-responsive hydrogel-based soft robot displayed a secondary 3D state at pH 11 and a primary 2D flat state in pH 2 butter solutions.

### 4.4. Pneumatic

**Figure 4:**
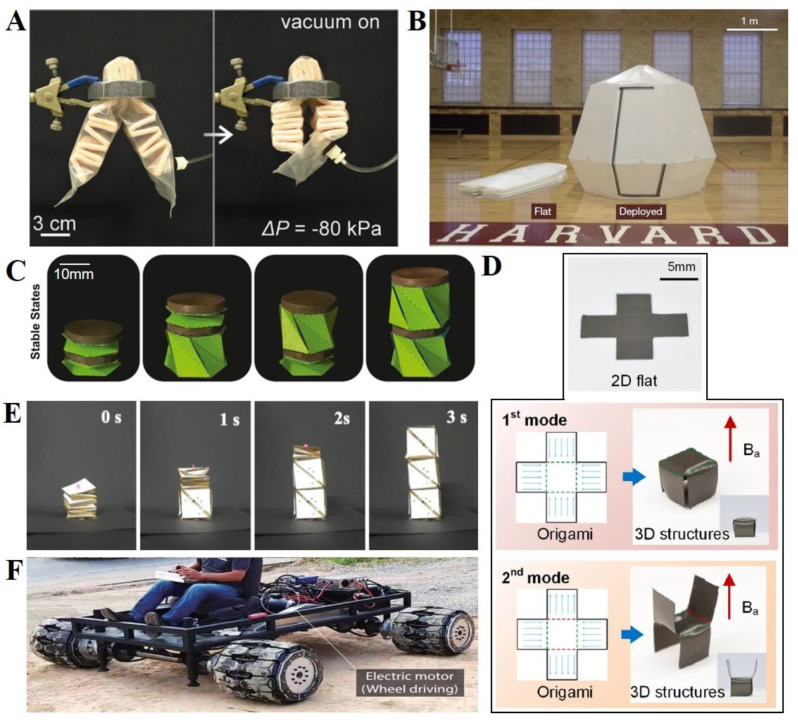
**Origami soft actuators.** (**A**) A soft linear pneumatic actuator combined with a metal screw nut. Reproduced with permission [122]. Adapted with permission under the terms of the Creative Commons Attribution Non-Commercial License 4.0, copyright 2017, the authors. (**B**) Pneumatic-driven multi-stable inflatable origami shelter. Reproduced with permission [48], copyright 2021, Springer Nature. (**C**) Schematics of the magnetic actuation of a two-cell Kresling assembly. Reproduced with permission [127], copyright 2020, PNAS. (**D**) Magnet field-responsive shape-morphing actuators. Reproduced with permission [128], copyright 2021, Elsevier Ltd. (**E**) Shape transformation of multilayered blocks combined with a torsional shape-memory alloy wire. Reproduced with permission [79], copyright 2020, IEEE. (**F**) A single-passenger vehicle for the installation of the transformable wheels. Reproduced with permission [137], copyright 2021, The American Association for the Advancement of Science.

The pneumatic actuation of origami soft robots has various advantages, such as easy implementation, large deformation, high energy efficiency, and low cost [122]. In general, pneumatic actuation can be divided into positive actuation [86,123,124,125] and vacuum actuation [73,122,126] according to the injection pressure. To actuate origami soft robots with pneumatic systems, the entire structure must be sealed [48,86,122,123]. For example, Li et al., presented an architecture for fluid-driven origami-inspired artificial muscles [122]. The artificial muscle is a compressible skeleton under vacuum pressure (Figure 4A). These muscles have a contraction ratio of 90%, a maximum stress of 600 kPa, and peak power densities of over 2 kW/kg. In addition, Melancon et al., presented a pneumatic-driven multi-stable inflatable origami structure [48]. Their proposed origami structures could maintain their inflatable shape without continuous actuation while ensuring multi-stability (Figure 4B). They demonstrated pressure-deployable origami structures based on an efficient geometry of triangular facets for inflatable and bistable structures. These triangular facets can be extended to several scale structures using proper fabrication, deployment, and stacking methods. However, it is difficult to expand these inflatable pneumatic structures to designs with many degrees of freedom. To overcome this limitation, many researchers have considered an advanced design concept of the modulation of soft origami robots that allows high degrees of freedom. For example, Lin et al., presented a vacuum-powered stiffness-controlled origami skeleton-shaped artificial muscle to achieve lightweight and multi-functionality. Each block consisted of sandpaper with origami creases and silicone rubber as the main body. A single block implements various motions; moreover, because it is simultaneously connected to the other blocks, its motion expands the structure. These pneumatic actuators have displayed the functionality of origami soft robots in terms of lifting, moving, and pipe-climbing [73]. Pneumatic control has the advantage of simply controlling the system; however, it is difficult to implement multifunctionality. However, this can only be implemented using a modular system. In general, the size of an implementable system is large, ranging from millimeters to meters.

### 4.5. Magnetic

In addition, magnetic control-based actuators have been extensively proposed at various scales [127,128,129,130,131,132,133]. Magnetic control-based actuators have the advantage of performing bistable motions by patterning the direction and intensity at magnetic fields to implement several motions in advance. In addition, magnetic actuators can be easily actuated with a fast response and can be wirelessly controlled in confined spaces. Novelino et al., proposed magnetic field-driven origami robots composed of a bistable Kresling pattern (Figure 4C) [127]. Kresling unit cells with magnetic-responsive plates can be triggered by following a bistable state transition with torsion. In their simulation model, any of the four stable states could operate correctly when two cells were connected via the ultrafast magnetic actuation method by controlling the applied magnetic field intensity and direction. Tang et al., presented a magnetically-driven actuator using the origami method by programming the direction of the magnetic moment of a soft actuator and adjusting the deformation width (Figure 4D) [128]. An actuator with shape-memory characteristics was fabricated by magnetizing a membrane with a specific origami structure by applying a uniform high-intensity magnetizing field and reconstructing a shape according to the origami structure using a relatively low external magnetic field. In particular, they designed a 3D self-driving rolling actuator that could contract its body up to 65% of its full length to pass through obstacles. The magnetic actuator can be adjusted to a small scale according to the magnetic field control, and is advantageous for distributed operations and complex shape implementation. However, as the size increases, the amount of equipment required for implementing the magnetic field may also increase.

### 4.6. Electronic

It is difficult to realize an active programmable origami device that exhibits large, fast, reversible, and stable movements in practical applications. To take full advantage of the deformability of origami structures, researchers have proposed diverse electronic-based SMA-based origami actuators [45,46,50,134,135,136,137,138]. Recently, Kim et al., presented a 3D shape-shifting actuator consisting of a reversibly morphing origami block with a torsional shape-memory alloy wire (TSW) actuated by electronics (Figure 4E) [50]. A pair of TSW actuators was embedded in the hinge lines of the origami actuators, which actively folded bidirectionally via the twisting motion of the SMA wire heated by passing an electric current through the wire. Each block unit can transform reversibly between a 2D flat sheet and 3D cubic block. Hawkes et al., proposed a programmable sheet that can be autonomously folded into different shapes [134]. In particular, the sheet, including all electronic circuitry, was folded into predetermined shapes using embedded SMAs. To generate a large torque from a thick (500 µm) sheet, the embedded SMAs were composed of relatively thin (100 µm) foil Nitinol SMAs [134]. These origami robot-based electronic actuators find it difficult to design miniaturized robots because of their essential electric components.

### 4.7. Motor

Furthermore, motor actuators can directly control origami robots by using mechanical energy transmission [51,139,140,141]. In general, motors usually provide rotational forces; therefore, it is necessary for a connected device to transform the driving forces into actuating origami robots [37,142,143]. Recently, Lee et al., reported a transformable wheel based on membrane origami that can withstand more than a 10-kN load (Figure 4F) [139]. An electric motor vehicle with hydraulic linear actuators can transform the wheel in approximately 5 s with a 1 m/s movement. Furthermore, Lee et al., presented a TWISTER hand, an under-actuated semi-soft gripper with a single servo motor. The finger was circular on the chassis and included a pulley mechanism for pulling and releasing cables from the fingers using a servo motor [142]. They found a relationship between the tensile force required to bend the structure and the contact force produced by the TWISTER mechanism to validate the cable-driven actuation. The motor actuator can drive the origami robot more stably, but there are problems with the size, noise, and vibration generated when the motor is operated.

## 5. Origami Design and Theory

Many simulation software packages, such as finite element analysis (FEA)-based ABAQUS (Daussault Systèmes SE, Paris, France), ANSYS (Ansys, Inc., Canonsburg, PA, USA), and COMSOL Multiphysics (COMSOL Inc., Burlington, MA, USA), have been widely adopted to analyze the material properties and geometric factors to understand the precise performance of origami soft robots [144]. By adopting these simulation software packages, diverse computational origami-shape reconfiguration studies have focused on structural deformation through folding simulations [74,145,146,147,148], flat state structure deformation according to the crease pattern [128,149,150,151], transformation by stimuli, such as pneumatic actuators [86,152,153], and energy efficiency according to the structure design [154].

**Figure 5:**
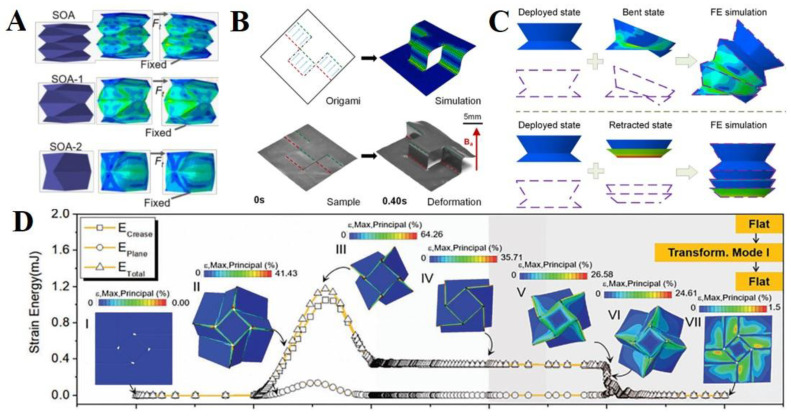
**Origami design and theory.** (**A**) Optimal design test of the Yoshimura origami-patterned tubes using the finite element method simulation. Reproduced with permission [74], copyright 2020, IEEE. (**B**) A set of complex 3D shape deformations of magnetic-driven actuators simulated by ABAQUS. Reproduced with permission [128], copyright 2021, Elsevier Ltd. (**C**) Shape prediction of the metawire by directly stacking the units with different shapes. Reproduced with permission [146], copyright 2021, Elsevier Ltd. (**D**) FEA results of the strain energy with mechanical folding from the initial flat state to transformation modes I and II, with a subsequent self-deployment process via thermal stimulus. Reproduced with permission [154], copyright 2020, WILEY-VCH.

Liu et al., obtained the optimal structure of an origami/kirigami approach to develop supernumerary robotic limbs driven by pneumatic input, including human limb functions, using a finite element method simulation in ABAQUS (Figure 5A) [74]. Specifically, a variation of the Yoshimura origami pattern was modeled to investigate the optimal design of a soft actuator with a high payload. This new approach for origami/kirigami patterns demonstrated unique advantages such as high strength, dexterity, and lighter weight in the design of wearable robotic devices. Tang et al., analyzed the deformation of magnetic-driven soft actuators used in ABAQUS (Figure 5B) [128]. First, they adapted a cantilever beam structure to verify the accuracy of infinite-element analysis and investigated the actuating mechanism of magnetized materials. The mechanism is a structural transformation based on a relatively low-intensity magnetic field obtained after manufacturing an actuator magnetized in a specific origami structure using a high-intensity magnetic field. They confirmed a mechanism for the bending distance and angle of the actuators based on magnetic field intensity and direction. Liu et al., also employed a finite element simulation using ABAQUS to analyze an energy-efficient morphing strategy. They used a meta wire that was serially stacked in each unit to realize a complex 3D target shape (Figure 5C) [146]. Each unit of the meta wire can be independently actuated for stretching, compression, and bending in a single direction. The simulation part was used to verify the independence of the meta wire when the number of units for the meta wire was increased, and it was deformed to obtain different shapes. Furthermore, Yap et al., developed a soft printable pneumatic actuator and analyzed the mechanical behavior of single- and dual-channel actuators with different input pressures [152]. Compared with the actual results of the injection pressure, the simulation results can predict the general bending behavior of the actuators at different input pressures using ABAQUS. Wang et al., developed a thermo-driven square-twist actuator to implement unique reconfigurable structures (Figure 5D) [154]. The square-twist structures are composed of rubbers for the crease area and thermal plastics for the rigid (plane) area. They simulated the changes in structural behavior when the geometry and material properties were varied using the implicit dynamic solver in ABAQUS. Specifically, two transformation modes were organized according to four parameters: the inner-side length, outside length, crease width, and crease thickness. Finally, the strain distribution according to each deformation structure were analyzed.

## 6. Applications

Numerous practical applications of 4D multiscale origami soft robots have been proposed, including locomotion robots [71,86,123,155,156,157,158,159], grippers [37,86,92,142], wheels [139], robotic arms [160], and exoskeletons [161,162]. In this section, we discuss diverse origami soft robots, including actuators, fluidic devices, biomedical grippers, and locomotion robots.

### 6.1. Actuators

Robotic actuators are generally developed based on the structural transformation with origami [90,143,163,164,165,166]. Kim et al., presented a soft fluidic bending actuator with gripping capability [166]. To compactly grip objects of various sizes, they constructed a dual-origami two-finger gripping unit comprising six modules. They designed four grippers—finger grip, suction grip, collaborative grip, and soft grippers—which can pick up objects of various sizes and materials. Recently, Ta et al., developed an origami jumper based on bistability with different body configurations [165]. The jumper was designed with a waterbomb pattern actuated by a phase-change liquid pouch actuator. A larger diameter makes the jumping force stronger; however, a large diameter is affected by air resistance. They positioned an origami jumper as a bio-inspired flexible and lightweight jumper. Kim et al., developed a self-locking robotic arm with a tendon-driven actuation system [143]. They designed an origami module with a self-locking mechanism and used seven modules to assemble a foldable arm using a gripper. The robotic arm is connected to drone and picks up objects during flight.

### 6.2. Biomedical Devices

**Figure 6:**
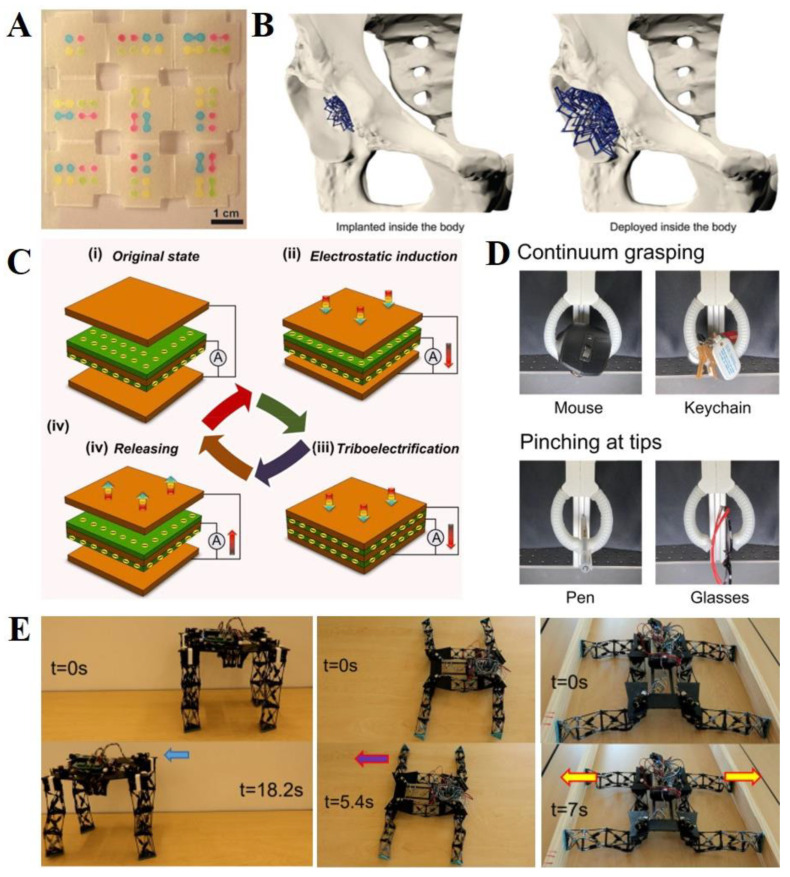
**Diverse origami soft robots.** (**A**) **Biomedical devices:** An unfolded, nine-layer paper micro-fluidic biosensor. Reproduced with permission [167], copyright 2011, American Chemical Society. (**B**) **Biomedical devices:** Origami design-based bone implant device. Reproduced with permission [168], copyright 2018, adapted with permission under the terms of the Creative Commons Attribution Non-Commercial License 3.0, copyright 2018, the authors. (**C**) **Energy harvester:** Origami-inspired triboelectric generator for ocean wave energy harvesting. Reproduced with permission [40], copyright 2019, Elsevier Ltd. (**D**) **Gripper:** An underactuated robotic gripper. Reproduced with permission [37], copyright 2021, IEEE. (**E**) **Soft robot:** Reconfigurable robot and its multiple modes of locomotion. Reproduced with permission [169], copyright 2022, IEEE.

From the perspective of actuators, origami soft robot designs can also be applied in many biomedical applications, including microfluidic biosensors and implant devices [9,10,29]. Liu et al., utilized origami principles to fabricate microfluidic biosensors for diagnostic purposes (Figure 6A) [167]. When the modules were folded into one rectangle, two analyte colorimetric assays of glucose and protein (bovine serum albumin, BSA) were conducted using just one injection, regardless of the number of layers. They specifically demonstrated that multilayer foldable microfluidic multichannel fabrication required just a few minutes to realize a cost-effective, convenient, and precise diagnosis. In addition, origami soft bio-implant machines have provided another solution for replacing lost biological tissue or actuating the real parts of our bodies. Recently, Bobbert et al., fabricated a deployable implant device using meta-biomaterials and origami designs (Figure 6B) [168]. These implant devices could display a less invasive size control system of minimal size before implantation and then increase in size later in the target zones. Therefore, they can reduce the risk of infection after implantation or damage to the surrounding area.

### 6.3. Other Applications: Energy Harvesting and Locomotion

Origami principles can also be used to develop effective energy-harvesting devices. Energy harvesting is a technology for collecting and storing external energy, such as solar or kinetic energy. Tao et al., proposed an origami-inspired triboelectric generator (TENG) for ocean wave energy harvesting (Figure 6C) [40]. TENG is a technology that converts the mechanical or thermal energy produced by small physical changes into electricity. The double helix spring structure based on the origami design has excellent elastic properties. Electrostatic induction and contact triboelectrification can be achieved by repeated compression and stretching of the generator. In addition, an underactuated gripper was presented for grasping complex objects using origami-inspired structures. Zou et al., presented stackable, pneumatic, actuator-based origami for an underactuated gripper [86]. Yu et al., developed an underactuated robotic gripper that could change grasping modes by two methods which can adjust the finger stiffness modulation (Figure 6D) [37]. These paper-based robots with highly deformable structures are similar to elastomer-based robots. In addition, an underwater gripper operating in the deep sea was developed for the nondestructive sampling of benthic fauna [92]. Finally, recent studies have presented locomotion robots with structural deformation to adjust the direction and angle (Figure 6E) [169]. Yang et al., proposed a manufacturing process for multi-chambered inflatable robots and structure-based origami [123]. Their proposed method was used to fabricate rolling and pipe-climbing robots. In addition, Luo et al., designed a snake robot with a cylindrical origami module structure driven by an electric motor and demonstrated lateral undulation and sidewinding locomotion modes [155].

## 7. Conclusions

In this review article, we analyze significant developments in versatile material selection, fabrication, and application in 4D multi-scale origami soft robots. Time-dependent 4D multi-scale origami soft robots have been widely highlighted as an innovative class of intelligent systems applicable to multi-functional actuators, grippers, and sensors. From the perspective of material selection, to obtain a new class of intelligent soft robotic systems, many soft materials, such as hydrogels, polymers, and hybrids, have been combined with functional materials, such as 2DLMs (e.g., graphene and MoS_2_) and nanomaterials (e.g., carbon nanotubes and liquid crystals). These hybrid materials exhibit extraordinary mechanical, electrical, chemical, and optical properties and can be utilized for realizing advanced smart 4D origami soft robots [170]. In terms of fabrication, various manufacturing strategies such as direct molding, casting, deploying, stacking, and 3D printing for shape reconfigurable multi-scale soft robots have been proposed over the past few decades. These active material and fabrication technologies have provided new capabilities and functionalities for 4D multi-scale origami soft robots. Origami-inspired shape transformable soft robots have demonstrated various technical opportunities applicable to smart manipulators (e.g., actuators and grippers), highly sensitive wearable electronics (e.g., sensors and batteries), and intelligent healthcare systems (e.g., grippers, surgeries, and drug deliveries).

Looking forward, several aspects must be addressed in shape transformation, locomotion, and functionalization of 4D multiscale origami soft robots. Untethered micro- or nanoscale structures are a major challenge in the manufacturing and operation of small-scale soft robots. In addition, the application of small-scale soft devices poses additional challenges in clinical practice (e.g., in vivo monitoring and imaging). For example, the precise navigation and transportation control of untethered small-scale soft robots is a significant challenge at deep in vivo locations for clinical drug delivery or microsurgery. Recently, ultrasound feedback coupled with gradient magnetic fields has demonstrated the possibility of realizing autonomous in vivo navigation and transportation in untethered small-scale soft robots [4]. Nevertheless, through the development of multidisciplinary research fields, 4D multiscale origami soft robots have provided new avenues for realizing intelligent soft robotic systems with multi-functional, multi-responsive, and highly sensitive feedback.

## Figures and Tables

**Table 1:** The 4D multiscale origami soft robots categorized based on materials, scales, and actuation triggers along with their advantages and disadvantages.

Materials	Scale	Advantages	Disadvantages	Actuation Triggers
Hydrogels	100 µm^−1^ cm	Suitable for untethered actuation.	Inapplicable for fabrication of large-scale robots because hydrogels give a localized response to external cues.	pH [34], Heat [34], Light [35]
Elastomers	10 mm^−5^ cm	Suitable for deformation of origami robots with self-recovery feature. Easy to actuate simple types of motions and tasks.	It is difficult to fix the folding pattern. Require relatively complex fabrication methods for folding.	Pneumatic [36], Motor [37]
Liquid Crystal	10 mm^−10^ cm	Suitable for actuators with direct control and deformation using photo-mechanisms.	Inapplicable for fabrication of large-scale robots because hydrogels give a localized response to external cues.	Heat [38], Light [39], Electric [40]
Shape-Memory Materials	500 µ^−10^ cm	High level of recoverable plastic strain (SMA). Applicable in several fields (SMP).	Not fit for application in medical-implantable devices (SMA). Low stress recovery and slow cycle time (SMP).	Heat [41,42], Electric [43,44,45,46], Moisture [44]
Hard Sheets	10 mm^−1^ m	Easy to fix folding crease pattern. Exhibit dramatic and versatile shape-changing capability. Lower cost.	Limitation of self-recovery for frequent deformation due to plastic deformation property.	Pneumatic [47,48], Magnetic [49], Electric [50], Motor [51]
